# Association between triglyceride glucose-body mass index and non-alcoholic fatty liver disease in the non-obese Chinese population with normal blood lipid levels: a secondary analysis based on a prospective cohort study

**DOI:** 10.1186/s12944-020-01409-1

**Published:** 2020-10-28

**Authors:** Yaling Li, Rui Zheng, Jie Li, Shuyi Feng, Li Wang, Zhiming Huang

**Affiliations:** 1 Department of Gastroenterology, The First Affiliated Hospital of Wenzhou Medical University, Wenzhou, 325000 Zhejiang Province China; 2 Department of Intensive Care Unit, The First Affiliated Hospital of Wenzhou Medical University, Wenzhou, 325000 Zhejiang Province China

**Keywords:** Insulin resistance, Triglyceride, Fasting plasma glucose, Non-alcoholic fatty liver disease, Body mass index, Triglyceride glucose body mass index, Secondary analysis, Association

## Abstract

**Background:**

Both triglyceride glucose-body mass index (TyG-BMI) and non-alcoholic fatty liver disease (NAFLD) are linked to insulin resistance (IR). Prospective studies linking TyG-BMI to NAFLD have been limited by short follow-up. This study investigated the longitudinal association between TyG-BMI and NAFLD occurrence in the non-obese Chinese individuals.

**Methods:**

This study determined TyG-BMI at baseline and the incidence of NAFLD at follow-up and performed a post hoc analysis of a prospective cohort study that involved assessing the risk of NAFLD in non-obese Chinese residents from January 2010 to December 2014. The incidence of NAFLD during the 5-year follow-up was identified as the endpoint. Cox proportional hazards regression analysis was used to evaluate hazard ratios (HRs) and 95% confidence intervals (95% CIs) for the incidence of NAFLD. Receiver operating characteristic (ROC) curve analysis was conducted to estimate the predictive power of TyG-BMI and its components for NAFLD. Subgroup analysis was performed to better understand other factors that may affect the association between TyG-BMI and NAFLD to identify potential special populations.

**Results:**

During the follow-up period, 841 (8.61%) of 9767 non-obese subjects who met the screening criteria were diagnosed with NAFLD. After confounding factors were fully adjusted for, the HR of NAFLD was 3.09 (95% CI 2.63–3.63) per standard deviation (SD) increase in TyG-BMI. Furthermore, TyG-BMI had a strong predictive value (area under ROC = 0.85; 95% CI 0.84–0.86) for the incidence of NAFLD, with a specificity of 0.73 and sensitivity of 0.82. Additionally, in the male population, each SD increase in TyG-BMI was linked to an increased risk of NAFLD (HR = 2.85, 95% CI 2.30–3.53), but the risk was higher in the female population (HR = 3.58, 95% CI 2.80–4.60). Gender and TyG-BMI interacted significantly with NAFLD incidence (*P* < 0.0001).

**Conclusion:**

In the normolipidaemic and non-obese subset of the Chinese population, an increase in TyG-BMI is related to an increased incidence of NAFLD. TyG-BMI may have clinical significance in identifying groups at high risk of NAFLD.

**Supplementary Information:**

The online version contains supplementary material available at 10.1186/s12944-020-01409-1.

## Introduction

Non-alcoholic fatty liver disease (NAFLD) has become a common form of chronic liver disease [1], and is closely related to type 2 diabetes and metabolic syndrome, with a prevalence between 18 and 45% [2, 3]. Regardless of whether there is underlying cirrhosis, NAFLD is considered to be the cause of hepatocellular carcinoma [4]. Therefore, early identification patients with a high risk of NAFLD is of great significance. NAFLD is more common among obese individuals than among nonobese individuals. Nevertheless, NAFLD has a prevalence rate of 3 to 30% in the non-obese population [5]. Several studies have shown that there is no significant difference in inflammation or fibrosis between non-obese NAFLD and obese NAFLD [6]. Dyslipidaemia is a well-known risk factor for NAFLD [7]. However, few studies have focused on the incidence of NAFLD in individuals with normal blood lipids [8]. A study by Sun et al. showed that increased normal low-density lipoprotein cholesterol (LDL-C) levels are associated with an elevated incidence of NAFLD [9]. Therefore, the risk of NAFLD merits attention even in people with normal blood lipids.

Although the underlying mechanism of NAFLD is unclear, insulin resistance (IR) is related to NAFLD development [10]. IR is considered to play an essential role in NAFLD pathogenesis in non-obese patients, regardless of the presence or absence of metabolic syndrome [11, 12]. The triglyceride and glucose (TyG) index, which combines fasting triglyceride (TG) and fasting plasma glucose (FPG), has been proposed as an effective substitute for IR [13]. Recently, triglyceride glucose body mass index (TyG-BMI), which combines TG, FPG, and obesity status, has been deemed more reliable than TyG for the identification of IR. The roles of all the parameters determining IR have been fully verified [14]. A cross-sectional study showed that TyG-BMI is linked to NAFLD [15]. However, it is not known whether the progression of time affects the association between TyG-BMI and NAFLD, and a subgroup analysis of gender has not been conducted, but previous studies have shown that gender affects the relationship [14]. Therefore, in this study, we attempted to collect 5-year longitudinal follow-up data from non-obese individuals with normal lipid profiles to investigate the association between TyG-BMI and NAFLD and explore whether gender affects the association between TyG-BMI and NAFLD.

## Methods

### Data source

The original data analysed were obtained from http://Datadryad.org, a public database that allows other investigators to reanalyse the data published by previous researchers. In keeping with the terms of service, this research cites data packets shared by Sun et al. [9, 16].

**Fig. 1:**
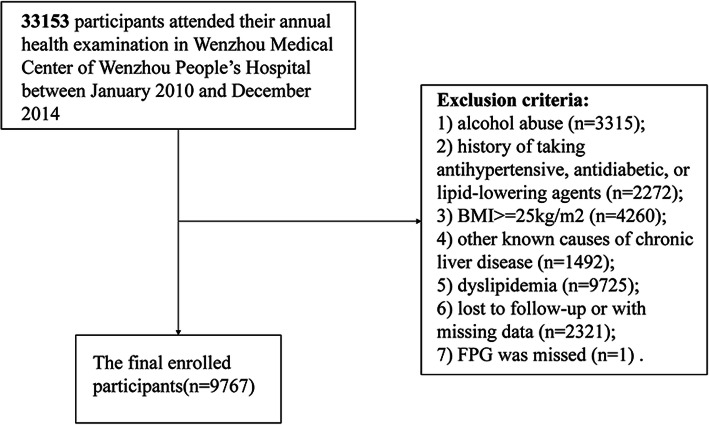
Flow chart

A post hoc analysis was conducted based on previous research that was a cohort study of 33,153 individuals enrolled at Wenzhou People’s Hospital from January 2010 to December 2014. All patients underwent a 5-year follow-up. The follow-up endpoint was the incidence of NAFLD. Subjects were assessed annually during the follow-up period. Subjects with the following conditions were excluded: (1) excessive drinking (> 70 g/week for females or 140 g/week for males) (*n* = 3315); (2) consumption of lipid-lowering, antidiabetic, or antihypertensive agents (*n* = 2272); (3) BMI ≥25 (*n* = 4260) [17]; (4) chronic liver disease caused by other factors (*n* = 1492); (5) dyslipidaemia [high-density lipoprotein cholesterol (HDL-C) < 1.04 mmol/L, LDL-C > 3.12 mmol/L, total cholesterol (TC) > 5.2 mmol/L, triglyceride (TG) > 1.7 mmol/L] (*n* = 9725); (6) loss to follow-up or missing information (*n* = 2321); and (7) missing FPG (*n* = 1) (shown in Fig. 1).

Because the data were de-identified, the requirement for informed consent was waived. The ethics committee of Wenzhou People’s Hospital had approved the previous study on which this one was based. Thus, this research did not require separate ethical approval. This study followed the Declaration of Helsinki.

### Data collection

As described in previous research, general clinical baseline data were collected by trained nurses through standardized patient-completed forms, and blood indicators were measured based on standard methods using an automated analyser (Abbott AxSYM) [18]. The following data were gathered: age, sex, systolic blood pressure (SBP), diastolic blood pressure (DBP), weight, height, total bilirubin (TB), direct bilirubin (DBIL), gamma-glutamyl transferase (GGT), alkaline phosphatase (ALP), globulin (GLB), aspartate aminotransferase (AST), alanine aminotransferase (ALT), albumin (ALB), total protein (TP), LDL-C, HDL-C, TG, TC, FPG, creatinine (Cr), blood urea nitrogen (BUN) and uric acid (UA). As introduced in the previous literature, TyG = Ln [TG (mg/dL) × FPG (mg/dL)/2] [19], and TyG-BMI = TyG × BMI [13].

### Diagnosis of NAFLD by ultrasound

NAFLD was diagnosed by ultrasound as described by the Chinese Liver Disease Association [18]. In short, NAFLD was defined as a diffusion-enhanced near-field echo in the liver region and gradual decay of the far-field echo in combination with one of the following conditions: the structure of the hepatic lacunae was not clearly displayed; mild to moderate hepatomegaly with peripheral and marginal passivation was present; the blood flow signal was reduced, but the blood flow distribution was normal; or the right liver lobe and diaphragm muscle capsule were unclear or incomplete [20].

### Missing data

For the 9767 participants in the analysed dataset, the Additional File Table S1 shows the missing data for each variable. For the missing covariates, multiple multivariate imputations were applied [21]. In order to minimize the bias that may be induced by excluding missing data from data analysis and maximizing statistical power [22], the Multiple Imputation by Chained Equations (MICE) software package was applied to create five estimated data sets with chained equations [21]. Additionally, as presented in Additional File Table S2, a sensitivity analysis was conducted to explore whether the resulting complete data were significantly different from the original data. The results indicated that the complete generated data were not significantly different from the original data. Thus, the multivariate analysis results were based on the original data set, as were all other analyses in this paper.

### Statistical analysis

All statistical analyses were conducted with EmpowerStats (www.empowerstats.com, X&Y Solutions, Inc., Boston, MA) and the statistical software package R (http://www.R-project.org, The R Foundation). Continuous variables that followed a normal distribution were expressed as the mean ± standard deviation (SD), and those that were not normally distributed were expressed as the median (quartile 1-quartile 3). Categorical variables were expressed as the frequency (percentage). The Mann-Whitney U test and chi-square test were employed as appropriate to evaluate the difference between NAFLD and non-NAFLD individuals. Results were considered statistically significant at a two-tailed *P* value of < 0.05.

The independent risk factors for NAFLD were determined by establishing univariate and multivariate Cox proportional hazard models. First, univariate analysis was performed to assess all variables, then, all variables that were statistically significant (*P* < 0.05) or regarded as clinically significant were included in the multivariate analysis. A correlation matrix was used to assess the collinearity of all explanatory variables. Collinearity between variables was tested using the variance inflation factor (VIF) based on a multiple regression model [23]. As shown in Additional File Table S3, the variables with VIF > 5 were considered to exhibit collinearity. Three different models were built: Model 1, with no adjustment for covariates; Model 2, adjusted for sex and age; and Model 3, adjusted for sex, age, ALP, LDL-C, HDL-C, UA, Cr, ALB, AST, ALT, GGT, GLB, FPG, TG, SBP, DBP, and DBIL.

A Cox proportional hazard model was generated for subgroup analysis. In the case of continuous variables, first, according to the clinical cut-off point or dichotomy, these variables were converted to categorical variables and then the interaction tests were performed. The subgroup effect modification test used the interaction terms between the subgroup indicators; then, likelihood ratio tests were carried out.

In order to verify the data analysis results and explore the possibility of nonlinearity, TyG-BMI was converted to categorical variables according to quartiles, and the *P* value for the trend was calculated. Receiver operating characteristic (ROC) curves were constructed to estimate the ability of TyG-BMI, TG, ALT, TyG, FPG, and BMI to predict NAFLD. In addition, the ratio of TG to HDL-C (TG/HDL-C) is reported to be associated with incident NAFLD [24]; accordingly, the ROC curve of TG/HDL-C was also drawn. The Kaplan-Meier method was applied to draw cumulative hazard curves and compare the cumulative incidence of NAFLD among the hierarchical TyG-BMI quartiles using the log-rank test.

## Results

### Description of the study groups

Of the 33,153 subjects recruited in the previous study, 9767 met the inclusion criteria for the present post hoc analysis (seen in Fig. 1). The subjects’ average age was 42.5 ± 14.7 (14–90) years, and 48.58% were women. Table 1 lists the baseline characteristics of the subjects. Individuals in the highest TyG-BMI group (Q4) were usually older and had higher BMI, LDL-C, TG, UA, FPG, Cr, TB, ALP, GGT, ALT, AST, GLB, SBP, DBP, TyG, TC, and BUN values than individuals in the lowest TyG-BMI group (Q1). In contrast, the ALB, HDL-C, and DBIL values of the Q3 and Q4 groups were lower than those of Q1. In addition, as TyG-BMI values increased, the incidence of NAFLD gradually increased (Q1: 0.29% vs. Q2: 1.43% vs. Q3: 7.58% vs. Q4: 25.14%).

**Table 1:** Baseline characteristics of subjects

	TyG-BMI				*P* value
	Q1 (114.23–157.67)	Q2 (157.68–172.11)	Q3 (172.11–188.07)	Q4 (188.08–230.41)	
**N**	2442	2441	2442	2442	
**Age**, years	41.28 ± 14.48	41.71 ± 14.19	43.20 ± 15.01	43.66 ± 15.03	< 0.001
**Sex**					< 0.001
Women	1243 (50.90%)	1264 (51.78%)	1185 (48.53%)	1053 (43.12%)	
Men	1199 (49.10%)	1177 (48.22%)	1257 (51.47%)	1389 (56.88%)	
**BMI**, kg/m^2^	18.56 ± 1.10	20.31 ± 0.87	21.75 ± 0.88	23.49 ± 0.89	< 0.001
**ALP**, U/L	64.16 ± 19.48	67.33 ± 23.42	70.88 ± 22.89	75.39 ± 22.12	< 0.001
**GGT**, U/L	17.00 (14.00–21.00)	17.00 (14.00–22.00)	20.00 (16.00–26.00)	24.00 (19.00–35.00)	< 0.001
**ALT**, U/L	13.00 (10.00–17.00)	14.00 (11.00–18.00)	15.00 (12.00–21.00)	19.00 (14.00–25.00)	< 0.001
**AST**, U/L	19.00 (17.00–22.00)	20.00 (17.00–23.00)	21.00 (18.00–25.00)	22.00 (19.00–26.00)	< 0.001
**TB**, μmol/L	12.22 ± 4.98	11.89 ± 4.71	12.18 ± 5.01	12.46 ± 4.97	0.012
**GLB**, g/L	29.35 ± 3.62	29.48 ± 3.73	29.53 ± 4.06	29.64 ± 4.05	0.104
**ALB**, g/L	44.48 ± 2.64	44.36 ± 2.75	44.21 ± 2.83	44.30 ± 2.78	0.009
**TP**, g/L	73.78 ± 3.97	73.77 ± 4.09	73.69 ± 4.19	73.89 ± 4.16	0.455
**Cr**, mmol/L	70.16 ± 21.11	73.21 ± 17.26	78.70 ± 26.06	86.05 ± 31.73	< 0.001
**UA**, μmol/L	233.34 ± 69.44	248.48 ± 72.94	270.44 ± 78.98	302.16 ± 80.37	< 0.001
**LDL-C**, mmol/L	1.97 ± 0.39	2.08 ± 0.40	2.16 ± 0.41	2.26 ± 0.41	< 0.001
**HDL-C**, mmol/L	1.61 ± 0.32	1.55 ± 0.29	1.49 ± 0.28	1.41 ± 0.26	< 0.001
**TG**, mmol/L	0.76 ± 0.23	0.90 ± 0.27	1.01 ± 0.28	1.20 ± 0.27	< 0.001
**TC**, mmol/L	4.23 ± 0.54	4.31 ± 0.53	4.37 ± 0.52	4.45 ± 0.50	< 0.001
**FPG**, mmol/L	4.85 ± 0.41	4.97 ± 0.45	5.09 ± 0.59	5.38 ± 1.04	< 0.001
**SBP**, mmHg	111.13 ± 13.65	114.95 ± 14.27	119.68 ± 15.66	126.49 ± 16.40	< 0.001
**DBP**, mmHg	67.74 ± 8.64	69.64 ± 9.31	72.09 ± 9.76	75.71 ± 10.22	< 0.001
**Time**, days	1036.08 ± 398.69	1012.46 ± 401.00	995.29 ± 394.25	925.52 ± 399.07	< 0.001
**TyG**	7.94 ± 0.31	8.13 ± 0.31	8.28 ± 0.30	8.50 ± 0.28	< 0.001
**BUN**, mmol/L	4.29 ± 1.36	4.42 ± 1.31	4.55 ± 1.36	4.74 ± 1.43	< 0.001
**DBIL**, μmol/L	2.46 ± 1.21	2.41 ± 1.28	2.39 ± 1.29	2.35 ± 1.17	0.136
**NAFLD**					< 0.001
No	2435 (99.71%)	2406 (98.57%)	2257 (92.42%)	1828 (74.86%)	
Yes	7 (0.29%)	35 (1.43%)	185 (7.58%)	614 (25.14%)	

The variables are presented as n (%) or the mean ± SD or median (quartile 1-quartile 3), TyG = Ln [TG (mg/dL) × FPG (mg/dL)/2], TyG-BMI = TyG × BMI

*Abbreviations*: *TyG-BMI* triglyceride glucose-body mass index, *BMI* body mass index, *NAFLD* non-alcoholic fatty liver disease, *TyG* triglyceride and glucose index, *DBP* diastolic blood pressure, *SBP* systolic blood pressure, *DBIL* direct bilirubin, *TB* total bilirubin, *GLB* globulin, *GGT* gamma-glutamyl transferase, *TP* total protein, *ALB* albumin, *UA* uric acid, *ALT* alanine aminotransferase, *AST* aspartate aminotransferase, *ALP* alkaline phosphatase, *Cr* creatinine, *BUN* blood urea nitrogen, *FPG* fasting plasma glucose, *TG* triglyceride, *TC* total cholesterol, *HDL-C* high-density lipoprotein cholesterol, *LDL-C* low-density lipoprotein cholesterol

### Predictive values of TyG-BMI for the incidence of NAFLD

**Fig. 2:**
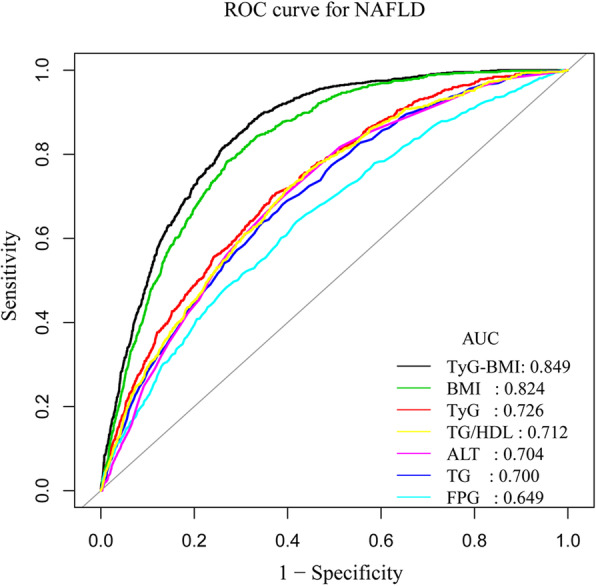
ROC curves for NAFLD

The ROC curve was plotted to measure the predictive power of TyG-BMI, TyG, BMI, ALT, TG, FPG and TG/HDL-C for NAFLD. Table 2 shows the predicted values of NAFLD. In ascending order, the predictive values of the variables for NAFLD were as follows: FPG [95% confidence interval (CI), 0.6291–0.6680; AUC (area under the curve) = 0.6485], TG (95% CI, 0.6829–0.7179; AUC = 0.7004), ALT (95% CI, 0.6858–0.7222; AUC = 0.7040), TG/HDL-C (95% CI, 0.6944–0.7288; AUC = 0.7116), TyG (95% CI, 0.7096–0.7433; AUC = 0.7264), BMI (95% CI, 0.8109–0.8361; AUC = 0.8235) and TyG-BMI (95% CI, 0.8375–0.8603; AUC = 0.8489). TyG-BMI predicted NAFLD with a specificity of 0.7348 and a sensitivity of 0.8205. The ROC curves of TyG-BMI and its components for predicting NAFLD are shown in Fig. 2. In addition, even when males and females were separated, TyG-BMI’s predictive ability for NAFLD was still better than that of other indicators (Additional File Fig. S1, S2).

**Table 2:** AUC of TyG-BMI, TyG, BMI, TG, FPG, ALT, and TG/HDL-C for predicting NAFLD

Variables	AUC	95% CI lower bound	95% CI upper bound	Best threshold	Specificity	Sensitivity
TyG-BMI	0.8489	0.8375	0.8603	183.8263	0.7348	0.8205
TyG	0.7264	0.7096	0.7433	8.3219	0.6325	0.7015
BMI	0.8235	0.8109	0.8361	22.1444	0.7308	0.7824
TG	0.7004	0.6829	0.7179	1.0450	0.6524	0.6397
FPG	0.6485	0.6291	0.6680	5.0350	0.5810	0.6397
ALT	0.7040	0.6858	0.7222	16.5000	0.6045	0.7047
TG/HDL-C	0.7116	0.6944	0.7288	0.6865	0.6053	0.7146

*Abbreviations*: *AUC* area under the curve, *CI* confidence interval

### Association between TyG-BMI and NAFLD

The results of the univariate analysis indicated that in the non-obese population, sex, age, DBP, SBP, BMI, LDL-C, HDL-C, TG, TC, FPG, UA, Cr, TB, GLB, ALB, AST, GGT, ALT, ALP, TP, BUN, DBIL, and TyG-BMI were crucial risk factors for NAFLD (Table 3). The effect sizes of the association between TyG-BMI and NAFLD incidence in females, males, and subjects in general are listed in Table 4. Model 1 is a crude model. This model showed that TyG-BMI was positively related to the incidence of NAFLD. In Model 2, for every 1-SD increase in TyG-BMI, the risk of NAFLD increased 4.046-fold (HR = 4.046, 95% CI 3.717–4.405, *P* < 0.001) after adjusting for sex and age. The fully adjusted HR (95% CI) for the incidence of NAFLD in all subjects was 3.089 (95% CI 2.628–3.631, *P* < 0.001) for every 1-SD increase in TyG-BMI. The fully adjusted HRs (95% CI) for women and men, respectively, were 3.583 (2.796, 4.593) and 2.849 (2.298, 3.532) .

**Table 3:** Univariate Cox regression model showing variables associated with NAFLD risk

Variables	Hazard ratio	Lower limit of 95% CI	Upper limit of 95% CI	*P* value
Sex (male)	1.127	0.984	1.29	0.085
Age	1.008	1.003	1.012	0.001
ALP	1.01	1.008	1.012	< 0.001
GGT	1.007	1.006	1.008	< 0.001
ALT	1.007	1.006	1.009	< 0.001
AST	1.013	1.007	1.018	< 0.001
TP	1.005	0.987	1.023	0.592
ALB	0.972	0.947	0.997	0.032
GLB	1.021	1.002	1.04	0.026
TB	0.988	0.972	1.004	0.131
Cr	1.005	1.004	1.006	< 0.001
UA	1.005	1.004	1.006	< 0.001
FPG	1.322	1.276	1.37	< 0.001
TC	1.398	1.221	1.601	< 0.001
TG	8.217	6.654	10.146	< 0.001
HDL-C	0.253	0.195	0.328	< 0.001
LDL-C	3.24	2.702	3.884	< 0.001
BMI	1.929	1.844	2.019	< 0.001
SBP	1.025	1.022	1.029	< 0.001
DBP	1.048	1.041	1.054	< 0.001
BUN	0.989	0.941	1.039	0.651
DBIL	0.59	0.54	0.646	< 0.001
TyG-BMI	1.071	1.067	1.076	< 0.001

**Table 4:** Association between TyG-BMI and NAFLD risk in different models

Exposure	Model 1	*P* value	Model 2	*P* value	Model 3	*P* value
	Hazard ratio (95% CI)		Hazard ratio (95% CI)		Hazard ratio (95% CI)	
**Female**						
TyG-BMI (1-SD increase)	4.170 (3.692, 4.709)	< 0.001	4.166 (3.688, 4.705)	< 0.001	3.583 (2.796, 4.593)	< 0.001
TyG-BMI Quartile						
Q1	1		1		1	
Q2	3.237 (1.186, 8.836)	0.022	3.237 (1.186, 8.836)	0.022	3.164 (0.669, 14.958)	0.146
Q3	19.206 (7.800, 47.293)	< 0.001	19.200 (7.797, 47.279)	< 0.001	10.716 (2.566, 44.744)	0.001
Q4	77.980 (32.206, 188.810)	< 0.001	77.920 (32.173, 188.713)	< 0.001	30.978 (7.427, 129.208)	< 0.001
*P* for trend	5.689 (4.762, 6.797)	< 0.001	5.687 (4.759, 6.797)	< 0.001	3.648 (2.733, 4.870)	< 0.001
**Male**						
TyG-BMI (1-SD increase)	3.954 (3.513, 4.451)	< 0.001	3.945 (3.504, 4.440)	< 0.001	2.849 (2.298, 3.532)	
TyG-BMI Quartile						
Q1	1		1		1	
Q2	9.985 (2.326, 42.868)	0.002	9.964 (2.321, 42.778)	0.002	8.338 (1.057, 65.749)	0.044
Q3	49.743 (12.264, 201.752)	< 0.001	49.323 (12.160, 200.065)	< 0.001	24.649 (3.369, 180.346)	0.002
Q4	171.006 (42.585, 686.699)	< 0.001	169.762 (42.273, 681.737)	< 0.001	56.284 (7.712, 410.750)	< 0.001
*P* for trend	5.098 (4.298, 6.047)	< 0.001	5.090 (4.291, 6.038)	< 0.001	3.098 (2.387, 4.020)	< 0.001
**Total**						
TyG-BMI (1-SD increase)	4.053 (3.724, 4.412)	< 0.001	4.046 (3.717, 4.405)	< 0.001	3.089 (2.628, 3.631)	< 0.001
TyG-BMI Quartile						
Q1	1		1		1	
Q2	5.164 (2.294, 11.626)	< 0.001	5.161 (2.292, 11.618)	< 0.001	4.718 (1.387, 16.040)	0.013
Q3	27.953 (13.142, 59.456)	< 0.001	27.832 (13.085, 59.202)	< 0.001	15.062 (4.743, 47.835)	< 0.001
Q4	104.387 (49.549, 219.914)	< 0.001	103.788 (49.261, 218.670)	< 0.001	38.242 (12.065, 121.213)	< 0.001
*P* for trend	5.387 (4.762, 6.094)	< 0.001	5.373 (4.749, 6.079)	< 0.001	3.336 (2.750, 4.047)	< 0.001

Model 1: unadjusted; Model 2: adjusted for sex and age; Model 3: adjusted for sex, age, ALP, GGT, ALT, AST, ALB, GLB, Cr, UA, FPG, TG, HDL-C, LDL-C, SBP, DBP, and DBIL

In order to explore the nonlinearity of TyG-BMI related to NAFLD events in patients, the continuous variable TyG-BMI was converted to categorical variables according to quartiles. The effect size trends of different TyG-BMI groups were equidistant, consistent with the *P* value for the trend of TyG-BMI for the occurrence of NAFLD in the patients (*P* < 0.001).

A sensitivity analysis was performed for imputation of missing covariates, and five imputed datasets were created by using the MICE software package with chained equations. When the same analysis was conducted in the five estimated data sets, the core results of the complete data analysis were stable and consistent with the original data (Additional File Table S2).

### Follow-up results

**Fig. 3:**
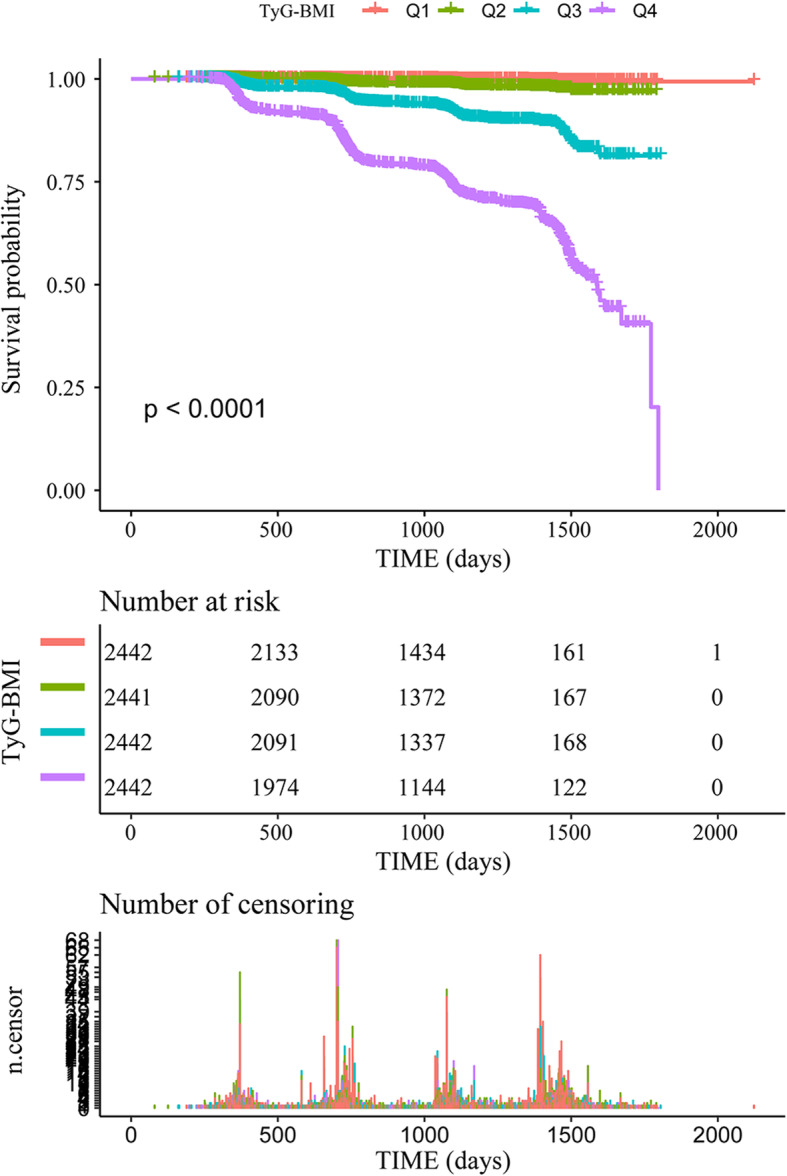
Kaplan-Meier analysis of NAFLD incidence according to TyG-BMI quartiles (*P* < 0.0001)

During the follow-up period, 841 (8.61%) of the non-obese subjects in the study were diagnosed with NAFLD. Figure 3 illustrates the significant difference in NAFLD risk between the TyG-BMI quartile groups (log-rank test *P* < 0.0001). As TyG-BMI increased, the cumulative risk of NALFD gradually increased.

### Subgroup analysis

**Fig. 4:**
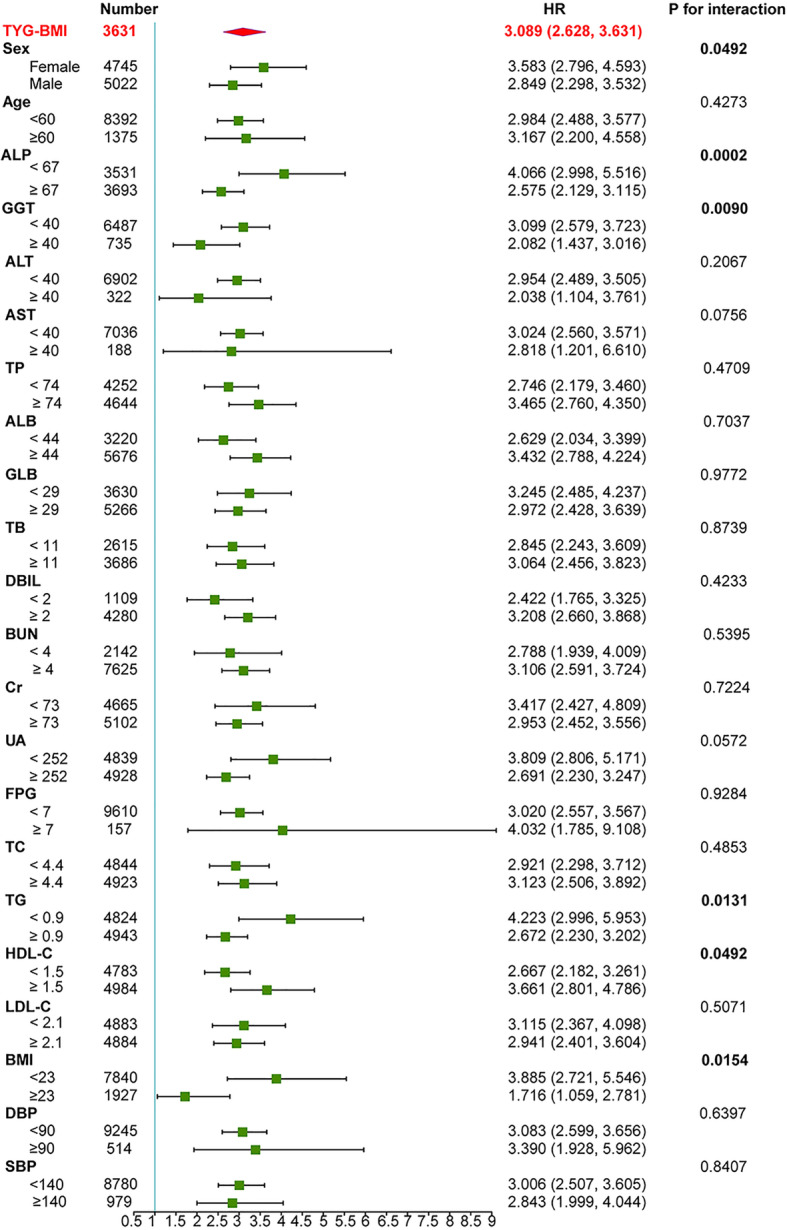
Subgroup analysis of the association between TyG-BMI and NAFLD. The HR (95% CI) was derived from the Cox regression model. (Sex, age, DBP, SBP, LDL-C, HDL-C, TG, UA, Cr, GLB, ALB, AST, ALP, GGT, ALT, FPG, and DBIL were adjusted)

In order to better understand other factors that may affect the association between TyG-BMI and NAFLD incidence and to further identify potential special populations, subgroup analysis was performed. The full variables were presented hierarchically based on clinical significance or bisection, and interaction tests were also performed (Additional File Table S4). The relationship between baseline TyG-BMI and NAFLD was weaker for men than for women (HR 2.849 versus HR 3.583, *P* < 0.05 for the TyG-BMI-gender interaction effect on NAFLD). The interactions of TyG-BMI with sex, ALP, GGT, HDL-C, TG, and BMI were significant. (shown in Fig. 4).

## Discussion

In this secondary analysis of a prospective cohort study, the association between TyG-BMI and non-obese NAFLD incidence in Chinese adults with normal lipid levels was explored. The results showed that in normal-weight individuals, TyG-BMI still showed a strong and positive association with NAFLD after adjusting for other covariates (HR = 3.089, 95% CI 2.628–3.631). Furthermore, TyG-BMI had a stronger predictive value than its components for the incidence of NAFLD, with larger AUC values in both genders. Therefore, TyG-BMI may be a valid indicator to predict the occurrence of NAFLD in non-obese Chinese individuals. As the Kaplan-Meier curves showed, the cumulative hazard of NAFLD gradually increased as TyG-BMI increased. Additionally, in the subgroup analysis, interaction effects on NAFLD risk were detected between TyG-BMI and sex (*P* value for interaction = 0.0492), ALP (*P* value for interaction = 0.0002), GGT (*P* value for interaction = 0.0090), TG (*P* value for interaction = 0.0131), HDL-C (*P* value for interaction = 0.0492) and BMI (*P* value for interaction = 0.0154).

Among non-obese people, NAFLD is not uncommon. Due to differences in study subjects’ choices, diagnostic methods, and lifestyles, the reported global prevalence of non-obese NAFLD ranges from 3 to 30% [25]. Kwon et al. [26] reported that among 29,994 subjects who underwent routine medical examinations, the prevalence of non-obese NAFLD was 12.6%. In a study from China, a total of 5562 participants completed a 5-year follow-up, of whom 494 (8.88%) developed non-obese NAFLD [7]. A study conducted in India reported that subjects with BMI < 23 kg/m^2^ and BMI < 25 kg/m^2^ had NAFLD prevalence rates of 5.5 and 7.4%, respectively [27]. Interestingly, in the stratified analysis, interactions (*P* value for interaction = 0.0154) were observed in individuals with BMI < 23 (HR = 3.885, 95% CI 2.721–5.546) and ≥ 23 (HR = 1.716, 95% CI 1.059–2.781). The relationship between baseline TyG-BMI and NAFLD was stronger in individuals with BMI < 23 than in those with higher BMI values. As shown in Additional File Table S4, when height and weight were devided into 3 groups each, an interaction effect of weight tertile and TyG-BMI on NAFLD risk was found (*P* value for interaction = 0.0154). Thus, the interaction with BMI may come from weight. Previous studies have reported that nicotine can reduce appetite and increase energy expenditure, leading to weight loss, and smoking can increase insulin resistance [28, 29], which may explain the interaction between body weight and TyG-BMI in this study. However, the raw data analysed in this study did not include smoking; therefore, further studies are still needed to verify this result.

Although the mechanism of NAFLD onset is poorly understood, IR is involved in NAFLD development [10]. Some studies have revealed that IR also plays an essential role in NAFLD pathogenesis in non-obese patients [11]. IR identification may help stratify non-obese NAFLD patients and support the development of personalized treatment measures. Nevertheless, the measurement of IR in clinical practice is not easy. The gold standard for testing IR is hyperinsulinaemic-euglycaemic clamp (HEC) [30], but this approach is time consuming and is not suitable for clinical application. Currently, the homeostasis model assessment of IR (HOMA-IR) is a universally accepted alternative indicator for IR. Previous studies have reported an independent association between NAFLD and HOMA-IR [31]. Additionally, other studies have suggested that HOMA-IR diagnostic criteria could be applied to predict NAFLD [32]. However, in many laboratories that perform routine tests, insulin measurement remains challenging, and there are problems with standardization [33]. Therefore, it is necessary to find more accessible and practical laboratory indicators.

Some studies have suggested that TyG could be used as an alternative index to assess IR [34, 35]. This parameter does not require a measurement of insulin concentration and requires only two simple indicators, namely, FPG and TG [13]. Moreover, TyG has been shown to be related to HOMA-IR and HEC by several studies and could be used to identify IR [36]. A study by Lee et al. [31] concluded that TyG performed better than HOMA-IR in predicting NAFLD. Regarding the role of obesity in IR, Er et al. and Lim et al. [13, 37] indicated that TyG-BMI performed better than TyG in predicting IR. Additionally, a study by Zeng et al. revealed that TyG-BMI performed better than TyG in identifying prehypertension in the non-obese population [14]. Zhang et al. [15] analysed the relationship between TyG-BMI and non-obese NAFLD in a cross-sectional study. They concluded that TyG-BMI was more effective than TyG alone in identifying non-obese NAFLD patients. The results of the present cohort study were consistent with theirs. However, their research has no follow-up data, and it is not known whether the passage of time affects the association between TyG-BMI and NAFLD. In addition, they did not consider the effect of gender on TyG, which was mentioned in previous articles [38]. In the present study, it was clearly observed that TyG-BMI was linked to the incidence of non-obese NAFLD in both genders. In male subjects, the TyG-BMI increase per SD was linked to an increased risk of NAFLD (HR = 2.85, 95% CI 2.30–3.53), but the risk was higher in female subjects (HR = 3.58, 95% CI 2.80–4.60). Gender and TyG-BMI had significant interactions with the occurrence of NAFLD (*P* < 0.0001). How gender affects the relationship between TyG-BMI and NAFLD is unclear, but it may be related to the effect of gender on glucose, lipid metabolism and IR [39, 40]. In addition, a protective effect of oestrogen against NAFLD has been suggested [41]; oestrogen levels drop in women after menopause, and subjects’ average age was 42.5 ± 14.7 years in this study, which may partly explain the higher HR of women in this study. Nevertheless, the specific mechanism requires further study.

### Strength and limitations of this study

This study has several strengths: (1) This was a 5-year longitudinal population-based study. (2) This study strictly adjusted for confounding factors. (3) The target independent variable was converted to a categorical variable for analysis, and both the complete data and the original data were analysed simultaneously to improve the reliability of the results. (4) Effect sizes were calculated in different populations.

Nevertheless, several limitations should be noted: (1) In this study, the diagnosis of NAFLD was based on ultrasonography rather than liver biopsy. The accuracy of NAFLD diagnosis may be reduced. In addition, ultrasonography cannot discriminate between steatosis and steatohepatitis. However, ultrasound examination for the diagnosis of NAFLD has been widely used in epidemiological studies [42]. (2) Some indicators associated with NAFLD and IR, such as waist circumference, waist-to-hip ratio, and HOMA-IR, were not collected in the raw data. (3) This study did not record information about energy intake and nutritional habits, but we indirectly adjusted for other covariates related to dietary habits, such as TC, HDL-C, LDL-C, and ALB. (4) Because the subjects included only non-obese people in China, the conclusion is not generalizable to other populations.

In short, the incidence of NAFLD without obesity is not uncommon in Chinese people. TyG-BMI is positively correlated with the incidence of NAFLD in non-obese people. In addition, the effect size may vary by gender. Thus, non-obese people with higher TyG-BMI need to be particularly concerned even if their blood lipids are at normal levels.

## Conclusion

In non-obese Chinese individuals with normal lipid levels, an increase in TyG-BMI is related to an increased incidence of NAFLD. TyG-BMI may have clinical significance in the early identification of groups with a high risk of NAFLD. This index is a simple and low-cost biochemical measurement value that may be used for large-scale NAFLD screening and risk assessment. Perhaps a strategy to prevent NAFLD can be developed based on the TyG-BMI index. Additionally, the findings of this study should be helpful for future research on the establishment of diagnostic or predictive models of incident NAFLD.

## Supplementary Information

**Additional File 1 Table S1**. Description of the missing data.

**Additional File 2 Table S2.** Comparative analysis of sensitivity before and after imputation.

**Additional File 3 Table S3**. Result of the collinearity test of each variable.

**Additional File 4 Table S4**. Subgroup analysis of the association between TyG-BMI and NAFLD risk.

**Additional File 5 Fig. S1**. ROC curves for NAFLD in males.

**Additional File 6 Fig. S2**. ROC curves for NAFLD in females.

## Data Availability

The data are available from the ‘DataDryad’ database (www.datadryad.org).

## Abbreviations

NAFLD: Non-alcoholic fatty liver disease
TyG-BMI: Triglyceride glucose body mass index
IR: Insulin resistance
AUC: Area under the curve
ROC: Receiver operating characteristic
HR: Hazard ratio
CI: Confidence interval
SD: Standard deviation
TC: Total cholesterol
TG: Triglyceride
SBP: Systolic blood pressure
DBP: Diastolic blood pressure
ALB: Albumin
GLB: Globulin
GGT: Gamma-glutamyl transferase
TP: Total protein
DBIL: Direct bilirubin
TB: Total bilirubin
UA: Uric acid
Cr: Creatinine
BUN: Blood urea nitrogen
ALT: Alanine aminotransferase
AST: Aspartate aminotransferase
ALP: Alkaline phosphatase
BMI: Body mass index
FPG: Fasting plasma glucose
HDL-C: High-density lipoprotein cholesterol
LDL-C: Low-density lipoprotein cholesterol
TyG: Triglyceride and glucose index
TG/HDL-C: Ratio of triglyceride to high-density lipoprotein cholesterol
VIF: Variance inflation factor
