# Supplementary material for: Association between triglyceride glucose-body mass index and non-alcoholic fatty liver disease in the non-obese Chinese population with normal blood lipid levels: a secondary analysis based on a prospective cohort study
Source: Lipids Health Dis. 2020 Oct 28;19:229. doi: 10.1186/s12944-020-01409-1 (PMC7592551; doi:10.1186/s12944-020-01409-1)
Supplement: Supplementary file 1 — Additional File 1 Table S1. Description of the missing data. [file 12944_2020_1409_MOESM1_ESM.docx]

**Additional File Table S1.docx: Description of the missing data.**

| Variables | Non-missing | missing |
| --- | --- | --- |
| age (years) | 9767 | 0 |
| sex | 9767 | 0 |
| ALP | 7224 | 2543 |
| GGT | 7222 | 2545 |
| ALT | 7224 | 2543 |
| AST | 7224 | 2543 |
| TP | 8896 | 871 |
| ALB | 8896 | 871 |
| GLB | 8896 | 871 |
| TB | 6301 | 3466 |
| DBIL | 5389 | 4378 |
| BUN | 9767 | 0 |
| Cr | 9767 | 0 |
| UA | 9767 | 0 |
| FPG | 9767 | 0 |
| TC | 9767 | 0 |
| TG | 9767 | 0 |
| HDL-C | 9767 | 0 |
| LDL-C | 9767 | 0 |
| BMI | 9767 | 0 |
| SBP | 9767 | 0 |
| DBP | 9767 | 0 |
| Time | 9767 | 0 |
| NAFLD | 9767 | 0 |
| TyG | 9767 | 0 |
| TyG-BMI | 9767 | 0 |
