# Supplementary material for: Association between triglyceride glucose-body mass index and non-alcoholic fatty liver disease in the non-obese Chinese population with normal blood lipid levels: a secondary analysis based on a prospective cohort study
Source: Lipids Health Dis. 2020 Oct 28;19:229. doi: 10.1186/s12944-020-01409-1 (PMC7592551; doi:10.1186/s12944-020-01409-1)
Supplement: Supplementary file 2 — Additional File 2 Table S2. Comparative analysis of sensitivity before and after imputation. [file 12944_2020_1409_MOESM2_ESM.docx]

**Additional File Table S2.docx: Comparative analysis of sensitivity before and after imputation.**

| Exposure | Model 1 | | Model 2 |  | Model 3 |  |
| --- | --- | --- | --- | --- | --- | --- |
|  | Hazard ratio (95% CI) | *P* value | Hazard ratio (95% CI) | *P* value | Hazard ratio (95% CI) | *P* value |
| **pre-imputation** |  |  |  |  |  |  |
| TyG-BMI  (1-SD increase) | 4.046 (3.717, 4.403) | <0.00001 | 4.046 (3.717, 4.405) | <0.00001 | 3.089 (2.628, 3.631) | <0.00001 |
| TyG-BMI Quartile |  |  |  |  |  |  |
| Q1 | 1 |  | 1 |  | 1 |  |
| Q2 | 5.166 (2.295, 11.630) | 0.00007 | 5.161 (2.292, 11.618) | 0.00007 | 4.718 (1.387, 16.040) | 0.01297 |
| Q3 | 27.942 (13.137, 59.432) | <0.00001 | 27.832 (13.085, 59.202) | <0.00001 | 15.062 (4.743, 47.835) | <0.00001 |
| Q4 | 104.178 (49.453, 219.463) | <0.00001 | 103.788 (49.261, 218.670) | <0.00001 | 38.242 (12.065, 121.213) | <0.00001 |
| *P* for trend | 5.378 (4.755, 6.083) | <0.00001 | 5.373 (4.749, 6.079) | <0.00001 | 3.336 (2.750, 4.047) | <0.00001 |
| **Input Data 1** |  |  |  |  |  |  |
| TyG-BMI  (1-SD increase) | 4.045 (3.717, 4.403) | <0.00001 | 4.046 (3.717, 4.405) | <0.00001 | 3.767 (3.355, 4.230) | <0.00001 |
| TyG-BMI Quartile |  |  |  |  |  |  |
| Q1 | 1.0 |  | 1.0 |  | 1.0 |  |
| Q2 | 5.160 (2.292, 11.617) | 0.00007 | 5.155 (2.290, 11.605) | 0.00007 | 4.474 (1.984, 10.086) | 0.00030 |
| Q3 | 27.926 (13.130, 59.399) | <0.00001 | 27.817 (13.077, 59.169) | <0.00001 | 20.223 (9.461, 43.231) | <0.00001 |
| Q4 | 104.121 (49.426, 219.342) | <0.00001 | 103.729 (49.233, 218.546) | <0.00001 | 60.654 (28.404, 129.524) | <0.00001 |
| *P* for trend | 5.378 (4.755, 6.083) | <0.00001 | 5.373 (4.749, 6.079) | <0.00001 | 4.266 (3.719, 4.894) | <0.00001 |
| **Input Data 2** |  |  |  |  |  |  |
| TyG-BMI  (1-SD increase) | 4.045 (3.717, 4.403) | <0.00001 | 4.046 (3.717, 4.405) | <0.00001 | 3.762 (3.349, 4.225) | <0.00001 |
| TyG-BMI Quartile |  |  |  |  |  |  |
| Q1 | 1.0 |  | 1.0 |  | 1.0 |  |
| Q2 | 5.160 (2.292, 11.617) | 0.00007 | 5.155 (2.290, 11.605) | 0.00007 | 4.468 (1.982, 10.073) | 0.00031 |
| Q3 | 27.926 (13.130, 59.399) | <0.00001 | 27.817 (13.077, 59.169) | <0.00001 | 20.262 (9.479, 43.313) | <0.00001 |
| Q4 | 104.121 (49.426, 219.342) | <0.00001 | 103.729 (49.233, 218.546) | <0.00001 | 60.481 (28.320, 129.162) | <0.00001 |
| *P* for trend | 5.378 (4.755, 6.083) | <0.00001 | 5.373 (4.749, 6.079) | <0.00001 | 4.257 (3.711, 4.885) | <0.00001 |
| **Input Data 3** |  |  |  |  |  |  |
| TyG-BMI  (1-SD increase) | 4.045 (3.717, 4.403) | <0.00001 | 4.046 (3.717, 4.405) | <0.00001 | 3.750 (3.340, 4.211) | <0.00001 |
| TyG-BMI Quartile |  |  |  |  |  |  |
| Q1 | 1.0 |  | 1.0 |  | 1.0 |  |
| Q2 | 5.160 (2.292, 11.617) | 0.00007 | 5.155 (2.290, 11.605) | 0.00007 | 4.519 (2.004, 10.188) | 0.00028 |
| Q3 | 27.926 (13.130, 59.399) | <0.00001 | 27.817 (13.077, 59.169) | <0.00001 | 20.281 (9.487, 43.356) | <0.00001 |
| Q4 | 104.121 (49.426, 219.342) | <0.00001 | 103.729 (49.233, 218.546) | <0.00001 | 60.917 (28.527, 130.083) | <0.00001 |
| *P* for trend | 5.378 (4.755, 6.083) | <0.00001 | 5.373 (4.749, 6.079) | <0.00001 | 4.266 (3.718, 4.894) | <0.00001 |
| **Input Data 4** |  |  |  |  |  |  |
| TyG-BMI  (1-SD increase) | 4.045 (3.717, 4.403) | <0.00001 | 4.046 (3.717, 4.405) | <0.00001 | 3.749 (3.339, 4.210) | <0.00001 |
| TyG-BMI Quartile |  |  |  |  |  |  |
| Q1 | 1.0 |  | 1.0 |  | 1.0 |  |
| Q2 | 5.160 (2.292, 11.617) | 0.00007 | 5.155 (2.290, 11.605) | 0.00007 | 4.497 (1.995, 10.139) | 0.00029 |
| Q3 | 27.926 (13.130, 59.399) | <0.00001 | 27.817 (13.077, 59.169) | <0.00001 | 20.356 (9.522, 43.515) | <0.00001 |
| Q4 | 104.121 (49.426, 219.342) | <0.00001 | 103.729 (49.233, 218.546) | <0.00001 | 60.999 (28.563, 130.272) | <0.00001 |
| *P* for trend | 5.378 (4.755, 6.083) | <0.00001 | 5.373 (4.749, 6.079) | <0.00001 | 4.269 (3.721, 4.898) | <0.00001 |
| **Input Data 5** |  |  |  |  |  |  |
| TyG-BMI  (1-SD increase) | 4.045 (3.717, 4.403) | <0.00001 | 4.046 (3.717, 4.405) | <0.00001 | 3.736 (3.326, 4.195) | <0.00001 |
| TyG-BMI Quartile |  |  |  |  |  |  |
| Q1 | 1.0 |  | 1.0 |  | 1.0 |  |
| Q2 | 5.160 (2.292, 11.617) | 0.00007 | 5.155 (2.290, 11.605) | 0.00007 | 4.446 (1.972, 10.023) | 0.00032 |
| Q3 | 27.926 (13.130, 59.399) | <0.00001 | 27.817 (13.077, 59.169) | <0.00001 | 20.116 (9.410, 43.001) | <0.00001 |
| Q4 | 104.121 (49.426, 219.342) | <0.00001 | 103.729 (49.233, 218.546) | <0.00001 | 59.928 (28.063, 127.978) | <0.00001 |
| *P* for trend | 5.378 (4.755, 6.083) | <0.00001 | 5.373 (4.749, 6.079) | <0.00001 | 4.246 (3.701, 4.871) | <0.00001 |
| **Combined result (Rubin’s)** |  |  |  |  |  |  |
| TyG-BMI  (1-SD increase) | 4.045 (3.717, 4.403) | <0.00001 | 4.046 (3.717, 4.405) | <0.00001 | 3.753 ( 3.344, 4.213) | <0.00001 |
| TyG-BMI Quartile |  |  |  |  |  |  |
| Q1 | 1 |  | 1 |  | 1 |  |
| Q2 | 5.160 (2.292, 11.617) | 0.00007 | 5.155 (2.290, 11.605) | 0.00007 | 4.481 (1.986, 10.079) | 0.00030 |
| Q3 | 27.926 (13.130, 59.399) | <0.00001 | 27.817 (13.077, 59.169) | <0.00001 | 20.248 (9.454, 43.113) | <0.00001 |
| Q4 | 104.121 (49.426, 219.342) | <0.00001 | 103.729 (49.233, 218.546) | <0.00001 | 60.596 (28.322, 129.324) | <0.00001 |
| *P* for trend | 5.378 (4.755, 6.083) | <0.00001 | 5.373 (4.749, 6.079) | <0.00001 | 4.257 (3.720, 4.890) | <0.00001 |

Model 1: unadjusted;
Model 2: adjusted for sex and age;
Model 3: adjusted for sex, age, ALP, GGT, ALT, AST, ALB, GLB, Cr, UA, FPG, TG, HDL-C, LDL-C, SBP, DBP, and DBIL.
