# Supplementary material for: Association between triglyceride glucose-body mass index and non-alcoholic fatty liver disease in the non-obese Chinese population with normal blood lipid levels: a secondary analysis based on a prospective cohort study
Source: Lipids Health Dis. 2020 Oct 28;19:229. doi: 10.1186/s12944-020-01409-1 (PMC7592551; doi:10.1186/s12944-020-01409-1)
Supplement: Supplementary file 3 — Additional File 3 Table S3. Result of the collinearity test of each variable. [file 12944_2020_1409_MOESM3_ESM.docx]

**Additional Files Table S3.docx: Result of the collinearity test of each variable.**

| Variables | VIF |
| --- | --- |
| TyG-BMI | 1.8 |
| Sex | 1.1 |
| Age | 1.1 |
| ALP | 1.2 |
| GGT | 1.3 |
| ALT | 3.0 |
| AST | 3.2 |
| ALB | 1.2 |
| GLB | 1.1 |
| TB | 2.2 |
| Cr | 1.6 |
| UA | 1.5 |
| FPG | 1.2 |
| TG | 1.6 |
| HDL-C | 1.3 |
| LDL-C | 1.1 |
| SBP | 2.5 |
| DBP | 2.0 |
| BUN | 1.6 |
| DBIL | 2.2 |

Abbreviation: VIF, variance inflation factor
