# Supplementary material for: Association between triglyceride glucose-body mass index and non-alcoholic fatty liver disease in the non-obese Chinese population with normal blood lipid levels: a secondary analysis based on a prospective cohort study
Source: Lipids Health Dis. 2020 Oct 28;19:229. doi: 10.1186/s12944-020-01409-1 (PMC7592551; doi:10.1186/s12944-020-01409-1)
Supplement: Supplementary file 4 — Additional File 4 Table S4. Subgroup analysis of the association between TyG-BMI and NAFLD risk. [file 12944_2020_1409_MOESM4_ESM.docx]

**Additional File Table S4. docx: Subgroup analysis of the association between TyG-BMI and NAFLD risk.**

| Characteristic | No. of participants | HR (95%CI) | *P* for interaction |
| --- | --- | --- | --- |
| Age (year) |  |  | 0.4273 |
| <60 | 8392 | 2.984 (2.488, 3.577) |  |
| ≥60 | 1375 | 3.167 (2.200, 4.558) |  |
| Sex |  |  | 0.0492 |
| Male | 5022 | 2.849 (2.298, 3.532) |  |
| Female | 4745 | 3.583 (2.796, 4.593) |  |
| ALP(U/L) |  |  | 0.0002 |
| <67 | 3531 | 4.066 (2.998, 5.516) |  |
| >=67 | 3693 | 2.575 (2.129, 3.115) |  |
| GGT(U/L) |  |  | 0.0090 |
| <40 | 6487 | 3.099 (2.579, 3.723) |  |
| >=40 | 735 | 2.082 (1.437, 3.016) |  |
| ALT(U/L) |  |  | 0.2067 |
| <40 | 6902 | 2.954 (2.489, 3.505) |  |
| >=40 | 322 | 2.038 (1.104, 3.761) |  |
| AST(U/L) |  |  | 0.0756 |
| <40 | 7036 | 3.024 (2.560, 3.571) |  |
| >=40 | 188 | 2.818 (1.201, 6.610) |  |
| TP(g/L) |  |  | 0.4709 |
| <74 | 4252 | 2.746 (2.179, 3.460) |  |
| >=74 | 4644 | 3.465 (2.760, 4.350) |  |
| ALB(g/L) |  |  | 0.7037 |
| <44 | 3220 | 2.629 (2.034, 3.399) |  |
| >=44 | 5676 | 3.432 (2.788, 4.224) |  |
| GLB(g/L) |  |  | 0.9772 |
| <29 | 3630 | 3.245 (2.485, 4.237) |  |
| >=29 | 5266 | 2.972 (2.428, 3.639) |  |
| TB(μmol/L) |  |  | 0.8739 |
| <11 | 2615 | 2.845 (2.243, 3.609) |  |
| >=11 | 3686 | 3.064 (2.456, 3.823) |  |
| DBIL(μmol/L) |  |  | 0.4233 |
| <2 | 1109 | 2.422 (1.765, 3.325) |  |
| >=2 | 4280 | 3.208 (2.660, 3.868) |  |
| BUN(mmol/L) |  |  | 0.5395 |
| <4 | 2142 | 2.788 (1.939, 4.009) |  |
| >=4 | 7625 | 3.106 (2.591, 3.724) |  |
| Cr(mmol/L) |  |  | 0.7224 |
| <73 | 4665 | 3.417 (2.427, 4.809) |  |
| >=73 | 5102 | 2.953 (2.452, 3.556) |  |
| UA(μmol/L) |  |  | 0.0572 |
| <252 | 4839 | 3.809 (2.806, 5.171) |  |
| >=252 | 4928 | 2.691 (2.230, 3.247) |  |
| FPG(mmol/L) |  |  | 0.9284 |
| <7 | 9610 | 3.020 (2.557, 3.567) |  |
| >=7 | 157 | 4.032 (1.785, 9.108) |  |
| TC(mmol/L) |  |  | 0.4853 |
| <4.39 | 4844 | 2.921 (2.298, 3.712) |  |
| >=4.39 | 4923 | 3.123 (2.506, 3.892) |  |
| TG(mmol/L) |  |  | 0.0131 |
| <0.93 | 4824 | 4.223 (2.996, 5.953) |  |
| >=0.93 | 4943 | 2.672 (2.230, 3.202) |  |
| HDL-C(mmol/L) |  |  | 0.0492 |
| <1.48 | 4783 | 2.667 (2.182, 3.261) |  |
| >=1.48 | 4984 | 3.661 (2.801, 4.786) |  |
| LDL-C(mmol/L) |  |  | 0.5071 |
| <2.14 | 4883 | 3.115 (2.367, 4.098) |  |
| >=2.14 | 4884 | 2.941 (2.401, 3.604) |  |
| BMI(kg/m2) |  |  | 0.0154 |
| <23 | 7840 | 3.885 (2.721, 5.546) |  |
| >=23 | 1927 | 1.716 (1.059, 2.781) |  |
| DBP(mmHg) |  |  | 0.6397 |
| <90 | 9245 | 3.083 (2.599, 3.656) |  |
| >=90 | 514 | 3.390 (1.928, 5.962) |  |
| SBP(mmHg) |  |  | 0.8407 |
| <140 | 8780 | 3.006 (2.507, 3.605) |  |
| >=140 | 979 | 2.843 (1.999, 4.044) |  |
| Weight ^Tertile^(kg) |  |  | 0.0154 |
| T1 (32.00-52.00) | 2793 | 5.455 (2.985, 9.971) |  |
| T2 (52.00-60.00) | 3528 | 4.043 (3.055, 5.351) |  |
| T3 (60.00-90.00) | 3446 | 2.796 (2.237, 3.493) |  |
| Height ^Tertile^(m2) |  |  | 0.8272 |
| T1 (1.34-1.60) | 3035 | 3.181 (2.419, 4.183) |  |
| T2 (1.60-1.67) | 3465 | 2.923 (2.319, 3.683) |  |
| T3 (1.67-1.97) | 3267 | 3.159 (2.543,3.926) |  |

Adjusted for sex, age, ALP, GGT, ALT, AST, ALB, GLB, Cr, UA, FPG, TG, HDL-C, LDL-C, SBP, DBP, DBIL.
