# Supplementary figures and images for: Association between triglyceride glucose-body mass index and non-alcoholic fatty liver disease in the non-obese Chinese population with normal blood lipid levels: a secondary analysis based on a prospective cohort study
Source: Lipids Health Dis. 2020 Oct 28;19:229. doi: 10.1186/s12944-020-01409-1 (PMC7592551; doi:10.1186/s12944-020-01409-1)

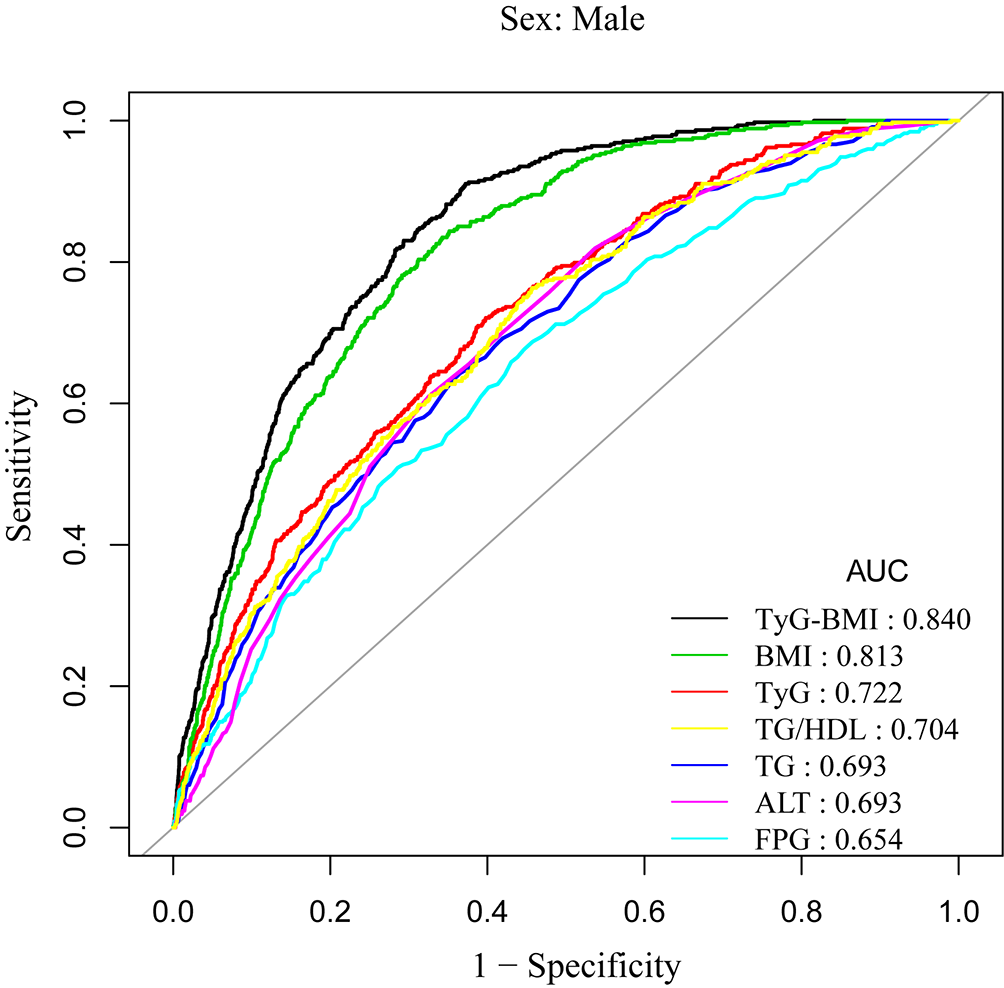

Supplement: Supplementary file 5 — Additional File 5 Fig. S1. ROC curves for NAFLD in males. [file 12944_2020_1409_MOESM5_ESM.tif]

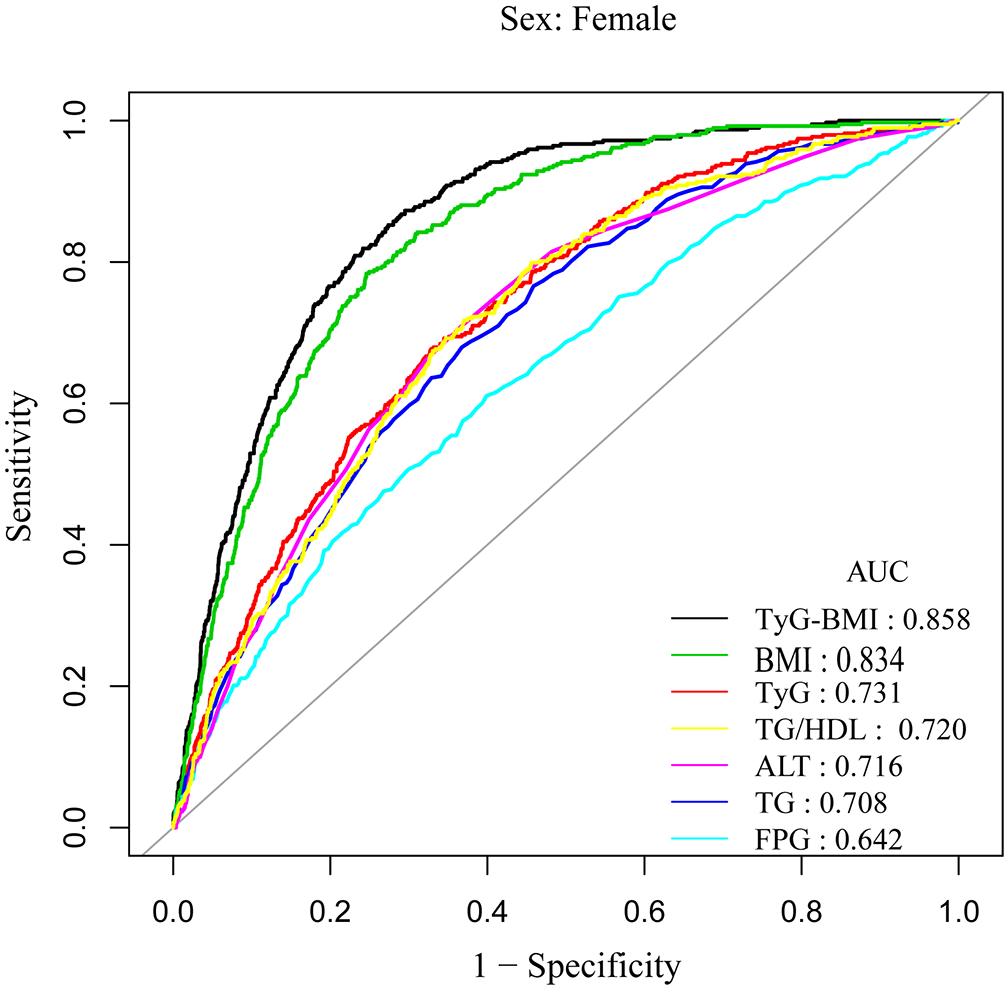

Supplement: Supplementary file 6 — Additional File 6 Fig. S2. ROC curves for NAFLD in females. [file 12944_2020_1409_MOESM6_ESM.tif]
